# Phase II study on early start of chemotherapy after excising primary colorectal cancer with distant metastases (Pearl Star 02)

**DOI:** 10.1002/ags3.12023

**Published:** 2017-08-14

**Authors:** Yoichiro Yoshida, Naoya Aisu, Daibo Kojima, Toshiyuki Mera, Fumiaki Kiyomi, Yuichi Yamashita, Suguru Hasegawa

**Affiliations:** ^1^ Department of Gastroenterological Surgery Fukuoka University Faculty of Medicine Fukuoka Japan; ^2^ Academia Industry and Government Collaborative Research Institute of Translational Medicine for Life Innovation Fukuoka University Fukuoka Japan

**Keywords:** chemotherapy, colorectal cancer, early start, surgery, XELOX

## Abstract

Initiating chemotherapy usually requires a delay of more than 4 weeks after surgically resecting colorectal cancer. However, there is little evidence regarding the required delay interval. We have previously reported a pilot study to determine the safety and feasibility of early initiation of chemotherapy after resecting primary colorectal cancer with distant metastases. We aimed to determine the safety and efficacy of early initiation of chemotherapy after resecting colorectal cancer with distant metastases.

This phase II study (trial number UMIN000006310) was a prospective, single‐arm trial. A total of 20 patients (men, 15 and women, 5) were enrolled. They underwent XELOX therapy (130 mg/m^2^ oxaliplatin on day 1+1000 mg/m^2^ capecitabine twice daily on days 1‐4) on postoperative day 7 and XELOX+bevacizumab (7.5 mg/kg bevacizumab on day 1) after the second chemotherapy cycle.

Baseline characteristics included a median age of 64 (range, 43‐72) years. Surgical procedures included right hemicolectomy in six patients, sigmoidectomy in three, anterior resection in five, and Hartmann procedure in six. All patients started chemotherapy on postoperative day 7. Median progression‐free survival was 14.9 months; overall response rate was 80%. Disease control rate was 100%. Grade 3 or higher hemotoxicity and grade 3 or higher non‐hematological toxicity was noted in 5.0% and 25.0% of patients, respectively. Postoperative complications were observed in two patients (superficial incisional surgical site infection and ileus).

Early initiation of chemotherapy after surgery is feasible. These findings suggest future changes of the start time of chemotherapy after surgery.

**Abbreviations**
CRC: colorectal cancer
XELOX: capecitabine and oxaliplatin
DCR: disease control rate
RR: objective tumor response rate
PFS: progression‐free survival
OS: overall survival
CR: complete response
PR: partial response
CI: confidence interval

## 1 INTRODUCTION

The National Comprehensive Cancer Network recommends that patients with metastatic colorectal cancer (CRC) undergo primary tumor resection if they have impending obstruction, bowel obstruction, or potentially resectable metastases. There is no doubt that resection or stoma placement is mandatory before starting systemic chemotherapy among patients with severe intestinal symptoms.^1^, ^2^, ^3^ Palliative resection of primary tumors reportedly improves systemic chemotherapy efficacy^4^ and prolongs time to treatment failure.^5^ In many cases, it is not possible for patients to continue chemotherapy because of complications, such as bleeding, perforation and bowel obstruction, if chemotherapy is started without surgical resection of the symptomatic primary tumor. Therefore, surgical resection of the primary tumor is apparently necessary to utilize chemotherapy with few complications. However, surgical resection may delay chemotherapy initiation.^6^ In general, a post‐surgical period longer than 4 weeks is standard until starting chemotherapy,^6^, ^7^ such as treatment with folinic acid, fluorouracil (5‐FU), and oxaliplatin; folinic acid, 5‐FU, and irinotecan; and capecitabine and oxaliplatin (XELOX). However, there is no positive evidence for this delay. In stage III disease, the time to start adjuvant therapy is an important prognostic factor for both colon and rectal cancers.^8^, ^9^, ^10^, ^11^ Early adjuvant therapy initiation is most often defined as starting therapy within 8 weeks after surgery, and it reportedly reduces the risks of recurrence and increases overall survival (OS) and disease‐free survival.^8^, ^9^ Metastatic tumors may rapidly enlarge before starting chemotherapy and may lead to patient death. It is unclear whether an even earlier initiation, such as within 1 week after surgery, may provide additional improvements. We conducted a clinical trial to prevent the early growth of metastatic lesions after primary resection. Because we previously reported that early initiation of chemotherapy after surgery is feasible,^12^ we evaluated its efficacy in patients subjected to colorectal surgery for symptomatic (narrowing of the stool, constipation, rectal bleeding, abdominal pain etc.) CRC with synchronous multiple distant metastases.

## 2 MATERIALS AND METHODS

### 2.1 Study design

Pearl Star 02 was a prospective, open‐label, single‐arm phase II trial that was completed in Japan. This study was carried out according to the ethical guidelines for clinical studies. The institutional review board at the Fukuoka University approved the protocol, and the study has been registered with the University Hospital Medical Information Network Clinical Trials Registry (ID: UMIN000006310).

This study evaluated the efficacy of early initiation of chemotherapy after resecting colorectal cancer with distant metastases. Primary endpoint was disease control rate (DCR), whereas secondary endpoints were objective tumor response rate (RR), progression‐free survival (PFS), overall survival (OS), and safety. The target sample size was 18 patients, assuming that the expected DCR and threshold DCR were 95% and 75%, respectively, with a one‐sided alpha level of 5% and a power of 80%. The expected DCR was decided based on past experience of the Pearl Star 01 trial at our hospital.^12^

### 2.2 Patients and eligibility criteria

In the present study, 20 patients were enrolled between September 2011 and June 2015. Eligibility criteria for selecting subjects were as follows: (i) age 20‐75 years; (ii) Eastern Cooperative Oncology Group performance status of 0 or 1; (iii) histologically confirmed CRC without prior chemo‐ or radiotherapy for metastatic disease; (iv) unresectable synchronous distant metastases; (v) adequate hematological (absolute leukocyte count, 4000‐12 000 leukocytes/mm^3^; neutrophil count, ≥1500 neutrophils/mm^3^; and platelet count, ≥100 000 platelets/mm^3^), hepatic (transaminase level, ≤100 IU/L and serum bilirubin level, ≤2.0 mg/dL), and renal (serum creatinine level, female: ≤1.35 mg/dL, male: ≤1.8 mg/dL) function; (vi) ability to take oral medications; and (vii) primary tumor resection 7 days before start of chemotherapy. Written informed consent was obtained from each patient.

Patients with any of the following conditions were excluded: history of serious hypersensitivity to drugs, active infection, symptomatic brain metastases, uncontrolled hypertension, uncontrolled diabetes, cirrhosis, clinically significant cardiovascular disease, history of myocardial infarction within the previous 3 months, uncontrolled angina pectoris or arrhythmia, multiple primary cancers within the past 5 years, pleural effusion requiring drainage, ascites or pericardial effusion, clinically significant mental or psychological disease, or any other condition that made the patient unsuitable for this study.

### 2.3 Treatment

Patients underwent XELOX therapy (130 mg/m^2^ oxaliplatin on day 1+1000 mg/m^2^ capecitabine twice daily on days 1‐14) on postoperative day 7 and XELOX+bevacizumab (7.5 mg/kg bevacizumab and 130 mg/m^2^ oxaliplatin on day 1+1000 mg/m^2^ capecitabine twice daily on days 1‐14, every 3 weeks) after the second chemotherapy cycle.^13^, ^14^ Dose reductions were required for all grade 3 or 4 toxicities that were attributed to the study medications. Treatment was continued until disease progression, unacceptable toxicities, or withdrawal of consent. Study treatment was delayed if any of the following criteria were applicable within 1 day of scheduled administration: neutrophil count, <1000/mm^3^; platelet count, <75 000/mm^3^; active infection with fever ≥38.0°C; grade 2 or worse diarrhea; grade 3 or worse peripheral sensory neurophathy (PSN); or other grade 2 or worse non‐hematological toxicities. Oxaliplatin dose was reduced to 100 mg/m^2^ if grade 3‐4 neutropenia or thrombocytopenia, persistent grade 2 or reversible grade 3 PSN, or any grade 3‐4 non‐hematological toxicities occurred. The patient was removed from the study if grade 3 toxicity persisted after a 21‐day washout period or if grade 4 PSN or grade 2‐4 allergic reaction occurred. The patient was also removed from the study if the patient required >3 weeks to recover from an adverse event.

### 2.4 Evaluation of chemotherapy

All patients underwent physical examination, chest radiography, and computed tomography of the abdomen, pelvis, and chest before start of treatment. All patients were included in the safety and efficacy analyses. Severity of adverse events was evaluated according to the National Cancer Institute Common Toxicity Criteria, version 4.0. Tumors were measured at 6‐ to 8‐week intervals, and responses were evaluated according to the Response Evaluation Criteria in Solid Tumors (RECIST), version 1.1. Evaluation of responses was based on radiologist‐reported measurements. Complete and partial responses required subsequent confirmation after an interval of at least 4 weeks. DCR was calculated from the number of patients who had a complete response (CR), partial response (PR), or stable disease with treatment, whereas RR was based on the number of patients who had either CR or PR. Progression was defined as objective tumor progression or death from any cause and was calculated using the Kaplan‐Meier method. PFS was calculated based on the date of study entry until progression or death. OS was calculated from the date of study entry until death from any cause and was calculated using the Kaplan‐Meier method. All calculations were carried out using SPSS IBM v. 23 (IBM, Chicago, IL, USA).

## 3 RESULTS

### 3.1 Baseline patient characteristics

Characteristics of the study patients are presented in Table 1. Twenty‐three patients fulfilled the inclusion criteria before surgery and three patients were excluded because of complications during the study period. No patients rejected informed consent. Baseline characteristics of the 15 male and five female participants included a median age of 64 (range, 43‐72) years. Eastern Cooperative Oncology Group (ECOG) performance status scores were 0 and 1 in 90.0% and 10.0% of patients, respectively. There was no emergency surgery. Right hemicolectomy was carried out in six patients, sigmoidectomy in three, anterior resection without stoma in five, and Hartmann procedure in six. Median operating time was 135 (range, 75‐292) min, and estimated blood loss was 98 (range, 5‐520) mL. Median follow‐up time was 24.3 months (range, 8.4 months to 44.0 months).

**Table 1:** Baseline characteristics of patients with colorectal cancer with distant metastases (N=20)

	N (%)	Median	Range
Age, years		64	43‐72
Gender			
Male	15 (75.0)		
Female	5 (25.0)		
ECOG Performance Status			
0	18 (90.0)		
1	2 (10.0)		
Primary cancer			
Colon	11 (55.0)		
Rectum	9 (45.0)		
Metastatic site			
Liver	18 (90.0)		
Lung	9 (45.0)		
Peritoneum	4 (20.0)		
Bone	1 (5.0)		
Operation			
Time (min)		135	75‐292
Bleeding (mL)		98	5‐520
Stoma (+)	6 (30)		
Stoma (−)	14 (70)		

ECOG, Eastern Cooperative Oncology Group.

### 3.2 Treatment

**Figure 1:**
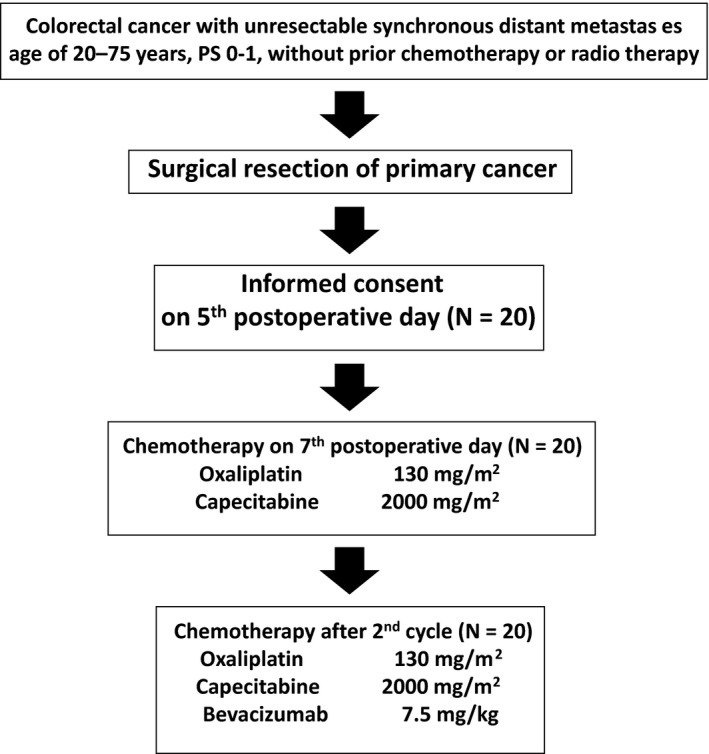
Flowchart of patients in the present study.

All patients successfully started chemotherapy on postoperative day 7 (Figure 1). Median number of chemotherapy cycles was 14 (range, 4‐30). Median cumulative dose of oxaliplatin was 1751 mg/m^2^. Fifteen patients (75.0%) continued treatment for more than eight cycles, whereas three discontinued treatment for adverse events and two discontinued treatment because of refusal or personal reasons. Four patients (20.0%) required dose reduction at least once within the eight cycles: two because of fatigue, one as a result of diarrhea, and one owing to thrombocytopenia.

### 3.3 Efficacy

**Figure 2:**
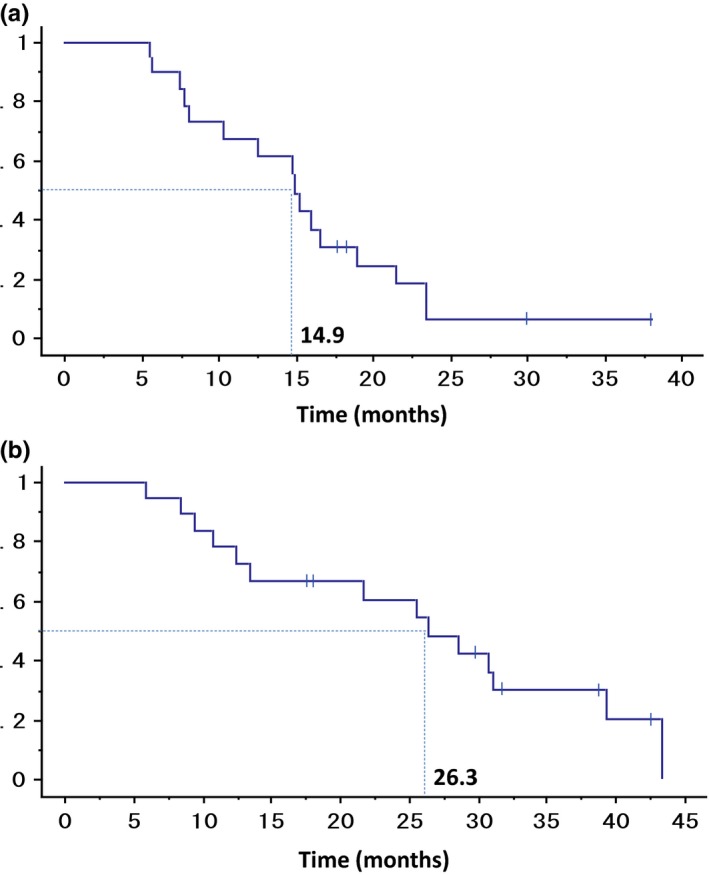
Kaplan‐Meier survival curves. (A) Progression‐free survival and (B) overall survival.

Confirmed response rate was 80.0% (95% confidence interval [CI], 56.3‐94.3) (CR, 0%; PR, 80.0%; stable disease, 20.0%; and progressive disease, 0%). DCR was 100%. PFS ranged from 5.5 to 38.2 months with a median of 14.9 months. OS was 26.3 months (Figure 2).

### 3.4 Safety

Adverse events for 20 patients are summarized in Table 2. During the postoperative period, postoperative complications were observed in two patients (superficial incisional surgical site infection [Clavien‐Dindo grade I] and ileus [Clavien‐Dindo grade I]). However, no patient developed complications because of starting chemotherapy, and no in‐hospital mortality occurred. One patient developed grade 2 thrombocytopenia. Grade 1 hand‐foot syndrome was observed in 65% of patients. Grade 3‐4 non‐hematological adverse events were reported in five patients.

**Table 2:** Hematological/non‐hematological adverse events and postoperative complications

Hematological grade 3‐4	Non‐hematological grade 3‐4
5.0%	25.0%
Thrombocytopenia: 1	Fatigue: 2
	Diarrhea: 1
	Pneumonia: 1
	Ileus: 1
Postoperative complications	
Superficial surgical site infection: 1	
Ileus: 1	

## 4 DISCUSSION

Surgical intervention has been suspected to promote cancer growth since ancient times but has received little attention from clinicians.^17^ Upfront primary tumor resection significantly increases the risk of progression for synchronous colorectal liver metastases as confirmed in a multivariate analysis.^18^, ^19^ Recent studies into the biology of metastasis formation and the tumor‐host relationship have added weight to the hypothesis of treatment‐induced stimulation of cancer growth and dissemination. The primary tumor and distant metastases are communicating ecosystems, which are characterized by a diversity of host cells that are both recruited from the bone marrow and locally.^20^ The ecosystems are also elucidated by the diversity of biological pathways responsible for the communication of the tumor cells with each of the host cells.^21^, ^22^

Plasma angiopoietin‐2 and vascular endothelial growth factor (VEGF) levels are significantly raised after CRC surgery.^23^ Peak levels were observed during days 7‐13 after surgery. The same researchers described that CRC surgery is linked to a persistent elevation in vascular cell adhesion molecule 1 (VCAM‐1) levels during the first month after surgery. VCAM‐1 may promote angiogenesis and chemotaxis of endothelial cells.^24^ Peeters et al.^25^ observed that metastasis growth is accompanied by increased cell proliferation and a significant decrease in the fraction of apoptotic cells on serial biopsies after resecting the primary tumor. The same researchers found significantly elevated cancer activity in liver metastases from CRC as measured by fluorodeoxyglucose‐positron emission tomography (FDG‐PET) after primary tumor resection.^18^

The precise timing for starting chemotherapy prior to and/or after surgery to avoid postoperative complications is unclear, but an interval of at least 4 weeks has been proposed. In most clinical trials, patients who underwent an operation within 4 weeks were excluded. We reported a case involving the early initiation of chemotherapy on postoperative day 7 who had undergone right hemicolectomy for synchronous multiple liver, lung, and peritoneal metastases.^26^ He survived without postoperative complications for 22 months despite huge liver metastases. Therefore, we carried out the present pilot study to confirm the feasibility of an immediate start for chemotherapy after surgery.^12^

Giving early postoperative 5‐FU has been investigated to improve outcomes after resecting intestinal malignancies.^27^ Surgeons have been reluctant to prescribe 5‐FU in the immediate postoperative period. This has primarily been associated with the belief that 5‐FU increases anastomotic leak rate, which could result in the need for reoperation, creation of a colostomy, need for a future takedown of colostomy, or even death. It has been estimated that one of every three postoperative deaths after colonic surgery was because of a leaking anastomosis.^28^ Several animal researchers have revealed that anastomoses were weaker and that there was a high risk of anastomotic leakage when systemic 5‐FU was given as a bolus immediately after surgery.^29^, ^30^ Immediate i.p. 5‐FU also causes anastomotic leakage.^31^ Continuous 5‐FU infusions enabled the use of greater daily dosages and appeared safer than injecting a bolus of 5‐FU.^32^ Continuous infusion avoided the high serum 5‐FU levels that could be observed with bolus injection dosage and may be efficacious in CRC without increasing anastomotic leakage. The oral fluoropyrimidine drug, capecitabine, evolved to ameliorate patient convenience and tolerability and has replaced continuous infusions of 5‐FU in many chemotherapy regimens.^33^ Capecitabine appears to be a promising substitute to continuous infusions of 5‐FU, and pharmacokinetic researchers have reported that successive oral administration provided a steady‐state 5‐FU concentration that was comparable with that achieved by a 5 day continuous infusion.^34^, ^35^ Furthermore, bolus 5‐FU treatments led to extremely high concentrations, followed by rapid disappearance from the blood serum.^34^, ^35^ Therefore, we chose XELOX therapy for the present study. Regarding safety, grade 3‐4 fatigue was observed in 10% of patients in this study. However, this result was more frequent than the 1‐3% of the SOFT study^36^ and the 4‐6% of the WJOG 4407G study.^37^ The reason may be because of synchronous metastases, early chemotherapy initiation, or adverse events of primary tumor resection.

According to recent reports,^38–50^ OS of patients with CRC and synchronous distant metastases was 11–21 months, whereas the previously reported PFS had ranged from 5.1 to 10.5 months (Table 3). Although our study cannot be compared with large multicenter trials, OS and PFS from this study were better than those from other studies. Therefore, early chemotherapy initiation after CRC surgery may prevent tumor growth. However, the results were obtained from the comparison with past reports using different regimens. This is a critical point for understanding the effect of early initiation of chemotherapy. Randomized controlled trials are considered essential to confirm the result. The primary CRC resection causing bleeding and severe stenosis is the first treatment step to prevent complications related to CRC. According to the Cochrane review, primary tumor resection is not associated with a consistent improvement in OS and fails to significantly reduce the risk of complications from the asymptomatic primary tumor^51^. However, our study enrolled symptomatic patients with CRC. The results will help identify answers and perform advanced trials.

**Table 3:** Comparison with other studies: Primary tumor resection with synchronous metastases

Author	Year	No. patients	Chemotherapy	PFS (95% CI)	OS (95% CI)
Tebutt et al.^38^	2003	280	5‐FU/raltitrexed+capecitabine	NA	14.0
Ruo et al.^39^	2003	127	NA	NA	16
Cook et al.^40^	2005	17658	NA	NA	Colon: 11
					Rectum: 16
Koopman et al.^41^	2007	258	Capecitabine/XELIRI	6.7	16.7
Galizia et al.^42^	2008	42	5‐FU±oxaliplatin/irinotecan	NA	17
Tol et al.^43^	2008	289	XELOX+bevacizumab+cetuximab	10.5	20.7
Bajwa et al.^44^	2009	32	5FU+oxaliplatin/5FU+oxaliplatin+irinotecan	NA	14
Evans et al.^45^	2009	45	NA	NA	11
Ferrand et al.^46^	2013	156	LV5FU2/pi5FU/raltitrexed	5.1 (4.6‐5.6)	16.3 (13.7‐19.2)
Ahmed et al.^47^	2014	761	5‐FU±oxaliplatin/irinotecan	NA	15.2
Tsang et al.^48^	2014	8599	NA	NA	21
Faron et al.^49^	2015	478	NA	NA	19.2 (18.2‐20.4)
Xu et al.^50^	2015	44514	NA	NA	16 (15.7‐16.3)
Present study	2016	20	XELOX+bevacizumab	14.9 (11.4‐18.4)	26.3 (20.6‐31.9)

CI, confidence interval; NA, not available; OS, overall survival; PFS, progression‐free survival.

To the best of our knowledge, this was the first report to determine the safety and efficacy of an early initiation of chemotherapy after resecting a primary CRC with distant synchronous metastases. Early initiation of chemotherapy after surgery may improve the prognosis of patients with CRC having synchronous metastases. Limitations include small numbers of patients and the nonrandomized study. Furthermore, this study included six patients without anastomosis Hartmann procedures. They were one‐third of all patients that was not small portion. Written informed consent was obtained from each patient on the 5^th^ postoperative day. Patients who had no postoperative complication were selected. Therefore, there is a risk for a considerable bias that we selected patients in an otherwise perfect state of health. Although we have shown that it was feasible to start chemotherapy a week after surgery, the actually benefit of this is uncertain. Randomized controlled trials will be essential to validate these findings. We might need more patients to clarify it.

## DISCLOSURE

Conflict of Interest: Authors declare no conflicts of interest for this article.

Author Contribution: Yo.Yo. designed and drafted the manuscript; S.H. provided analytical oversight; Yu.Ya. revised the manuscript for important intellectual content; N.A., D.K., T.M., S.H. provided administrative support; F.K. did the statistical analysis. All authors have read and approved the final version to be published.

## References

[1] Joffe J , Gordon PH . Palliative resection for colorectal carcinoma. Dis Colon Rectum. 1981;24:355–60. 6167412 10.1007/BF02603417

[2] Longo WE , Ballantyne GH , Bilchik AJ , et al. Advanced rectal cancer. What is the best palliation? Dis Colon Rectum. 1988;31:842–7. 2460299 10.1007/BF02554846

[3] Rosen SA , Buell JF , Yoshida A , et al. Initial presentation with stage IV colorectal cancer: how aggressive should we be? Arch Surg. 2000;135:530–4; discussion 534‐5. 10807276 10.1001/archsurg.135.5.530

[4] Temple LK , Hsieh L , Wong WD , et al. Use of surgery among elderly patients with stage IV colorectal cancer. J Clin Oncol. 2004;22:3475–84. 15337795 10.1200/JCO.2004.10.218

[5] Kaufman MS , Radhakrishnan N , Roy R , et al. Influence of palliative surgical resection on overall survival in patients with advanced colorectal cancer: a retrospective single institutional study. Colorectal Dis. 2008;10:498–502. 17949445 10.1111/j.1463-1318.2007.01384.x

[6] Benoist S , Pautrat K , Mitry E , et al. Treatment strategy for patients with colorectal cancer and synchronous irresectable liver metastases. Br J Surg. 2005;92:1155–60. 16035135 10.1002/bjs.5060

[7] Boselli C , Renzi C , Gemini A , et al. Surgery in asymptomatic patients with colorectal cancer and unresectable liver metastases: the authors’ experience. OncoTargets Ther. 2013;6:267. 10.2147/OTT.S39448 PMC3615897 23569390

[8] Biagi JJ , Raphael MJ , Mackillop WJ , et al. Association between time to initiation of adjuvant chemotherapy and survival in colorectal cancer: a systematic review and meta‐analysis. JAMA. 2011;305:2335–42. 21642686 10.1001/jama.2011.749

[9] Yu S , Shabihkhani M , Yang D , et al. Timeliness of adjuvant chemotherapy for stage III adenocarcinoma of the colon: a measure of quality of care. Clin Colorectal Cancer. 2013;12:275–9. 24188686 10.1016/j.clcc.2013.08.002

[10] Tevis SE , Kohlnhofer BM , Stringfield S , et al. Postoperative complications in patients with rectal cancer are associated with delays in chemotherapy that lead to worse disease‐free and overall survival. Dis Colon Rectum. 2013;56:1339–48. 24201387 10.1097/DCR.0b013e3182a857eb PMC3884512

[11] Cheung WY , Neville BA , Earle CC . Etiology of delays in the initiation of adjuvant chemotherapy and their impact on outcomes for Stage II and III rectal cancer. Dis Colon Rectum. 2009;52:1054–63; discussion 1064. 19581846 10.1007/DCR.0b013e3181a51173

[12] Yoshida Y , Hoshino S , Aisu N , et al. Pilot study of the early start of chemotherapy after resection of primary colorectal cancer with distant metastases (Pearl Star 01). World J Surg Oncol. 2013;11:39. 23388092 10.1186/1477-7819-11-39 PMC3570337

[13] Yoshida Y , Hoshino S , Aisu N , et al. Administration of chemotherapy via the median cubital vein without implantable central venous access ports: port‐free chemotherapy for metastatic colorectal cancer patients. Int J Clin Oncol. 2015;20:332–7. 24811333 10.1007/s10147-014-0703-5

[14] Yoshida Y , Hirata K , Matsuoka H , et al. A single‐arm Phase II validation study of preventing oxaliplatin‐induced hypersensitivity reactions by dexamethasone: the AVOID trial. Drug Des Devel Ther. 2015;9:6067–73. 10.2147/DDDT.S94901 PMC4648596 26648694

[15] US Department of Health and Human Services National Institutes of Health National Cancer Institute . Common Terminology Criteria for Adverse Events (CTCAE) Version 4.0., US Department of Health and Human Services National Institutes of Health National Cancer Institute; 2009.

[16] Eisenhauer EA , Therasse P , Bogaerts J , et al. New response evaluation criteria in solid tumours: revised RECIST guideline (version 1.1). Eur J Cancer. 2009;45:228–47. 19097774 10.1016/j.ejca.2008.10.026

[17] Coffey JC , Wang JH , Smith MJ , et al. Excisional surgery for cancer cure: therapy at a cost. Lancet Oncol. 2003;4:760–8. 14662433 10.1016/s1470-2045(03)01282-8

[18] Scheer MG , Stollman TH , Vogel WV , et al. Increased metabolic activity of indolent liver metastases after resection of a primary colorectal tumor. J Nucl Med. 2008;49:887–91. 18483084 10.2967/jnumed.107.048371

[19] Slesser AA , Khan F , Chau I , et al. The effect of a primary tumour resection on the progression of synchronous colorectal liver metastases: an exploratory study. Eur J Surg Oncol. 2015;41:484–92. 25638603 10.1016/j.ejso.2014.12.009

[20] Ceelen W , Pattyn P , Mareel M . Surgery, wound healing, and metastasis: recent insights and clinical implications. Crit Rev Oncol Hematol. 2014;89:16–26. 23958676 10.1016/j.critrevonc.2013.07.008

[21] Mareel M , Oliveira MJ , Madani I . Cancer invasion and metastasis: interacting ecosystems. Virchows Arch. 2009;454:599–622. 19471961 10.1007/s00428-009-0784-0

[22] Viani GA , Stefano EJ , Soares FV , et al. Evaluation of biologic effective dose and schedule of fractionation for preoperative radiotherapy for rectal cancer: meta‐analyses and meta‐regression. Int J Radiat Oncol Biol Phys. 2011;80:985–91. 20615619 10.1016/j.ijrobp.2010.03.008

[23] Kumara HM , Feingold D , Kalady M , et al. Colorectal resection is associated with persistent proangiogenic plasma protein changes: postoperative plasma stimulates in vitro endothelial cell growth, migration, and invasion. Ann Surg. 2009;249:973–7. 19474682 10.1097/SLA.0b013e3181a6cd72

[24] Shantha Kumara HM , Tohme ST , Herath SA , et al. Plasma soluble vascular adhesion molecule‐1 levels are persistently elevated during the first month after colorectal cancer resection. Surg Endosc. 2012;26:1759–64. 22219007 10.1007/s00464-011-2112-4

[25] Peeters CF , de Waal RM , Wobbes T , et al. Outgrowth of human liver metastases after resection of the primary colorectal tumor: a shift in the balance between apoptosis and proliferation. Int J Cancer. 2006;119:1249–53. 16642475 10.1002/ijc.21928

[26] Yoshida Y , Hoshino S , Shiwaku H , et al. Early start of chemotherapy after resection of primary colon cancer with synchronous multiple liver metastases: a case report. Case Rep Oncol. 2011;4:250–4. 21677890 10.1159/000328805 PMC3104872

[27] van der Kolk BM , de Man BM , Wobbes T , et al. Is early post‐operative treatment with 5‐fluorouracil possible without affecting anastomotic strength in the intestine? Br J Cancer. 1999;79:545–50. 10027328 10.1038/sj.bjc.6690086 PMC2362429

[28] Debas HT , Thomson FB . A critical review of colectomy with anastomosis. Surg Gynecol Obstet. 1972;135:747–52. 5084246

[29] Goldman LI , Lowe S , al‐Saleem T , Effect of fluorouracil on intestinal anastomoses in the rat. Arch Surg. 1969;98:303–4. 5766277 10.1001/archsurg.1969.01340090079011

[30] Morris T . Retardation of healing of large‐bowel anastomoses by 5‐fluorouracil. Aust N Z J Surg. 1979;49:743–5. 294271

[31] Weiber S , Graf W , Glimelius B , et al. Experimental colonic healing in relation to timing of 5‐fluorouracil therapy. Br J Surg. 1994;81:1677–80. 7827906 10.1002/bjs.1800811140

[32] Lokich JJ , Ahlgren JD , Gullo JJ , et al. A prospective randomized comparison of continuous infusion fluorouracil with a conventional bolus schedule in metastatic colorectal carcinoma: a Mid‐Atlantic Oncology Program Study. J Clin Oncol. 1989;7:425–32. 2926468 10.1200/JCO.1989.7.4.425

[33] Bennouna J , Saunders M , Douillard JY . The role of UFT in metastatic colorectal cancer. Oncology. 2009;76:301–10. 19299903 10.1159/000209334

[34] Ho DH , Pazdur R , Covington W , et al. Comparison of 5‐fluorouracil pharmacokinetics in patients receiving continuous 5‐fluorouracil infusion and oral uracil plus N1‐(2′‐tetrahydrofuryl)‐5‐fluorouracil. Clin Cancer Res. 1998;4:2085–8. 9748123

[35] Borner MM , Schoffski P , de Wit R , et al. Patient preference and pharmacokinetics of oral modulated UFT versus intravenous fluorouracil and leucovorin: a randomised crossover trial in advanced colorectal cancer. Eur J Cancer. 2002;38:349–58. 11818199 10.1016/s0959-8049(01)00371-9

[36] Yamada Y , Takahari D , Matsumoto H , et al. Leucovorin, fluorouracil, and oxaliplatin plus bevacizumab versus S‐1 and oxaliplatin plus bevacizumab in patients with metastatic colorectal cancer (SOFT): an open‐label, non‐inferiority, randomised phase 3 trial. Lancet Oncol. 2013;14:1278–86. 24225157 10.1016/S1470-2045(13)70490-X

[37] Yamazaki K , Nagase M , Tamagawa H , et al. Randomized phase III study of bevacizumab plus FOLFIRI and bevacizumab plus mFOLFOX6 as first‐line treatment for patients with metastatic colorectal cancer (WJOG4407G). Ann Oncol. 2016;27:1539–46. 27177863 10.1093/annonc/mdw206

[38] Tebbutt NC , Norman AR , Cunningham D , et al. Intestinal complications after chemotherapy for patients with unresected primary colorectal cancer and synchronous metastases. Gut. 2003;52:568–73. 12631671 10.1136/gut.52.4.568 PMC1773619

[39] Ruo L , Gougoutas C , Paty PB , et al. Elective bowel resection for incurable stage IV colorectal cancer: prognostic variables for asymptomatic patients. J Am Coll Surg. 2003;196:722–8. 12742204 10.1016/S1072-7515(03)00136-4

[40] Cook AD , Single R , McCahill LE . Surgical resection of primary tumors in patients who present with stage IV colorectal cancer: an analysis of surveillance, epidemiology, and end results data, 1988 to 2000. Ann Surg Oncol. 2005;12:637–45. 15965730 10.1245/ASO.2005.06.012

[41] Koopman M , Antonini NF , Douma J , et al. Sequential versus combination chemotherapy with capecitabine, irinotecan, and oxaliplatin in advanced colorectal cancer (CAIRO): a phase III randomised controlled trial. Lancet. 2007;370:135–42. 17630036 10.1016/S0140-6736(07)61086-1

[42] Galizia G , Lieto E , Orditura M , et al. First‐line chemotherapy vs bowel tumor resection plus chemotherapy for patients with unresectable synchronous colorectal hepatic metastases. Arch Surg. 2008;143:352–8; discussion 358. 18427022 10.1001/archsurg.143.4.352

[43] Tol J , Koopman M , Rodenburg CJ , et al. A randomised phase III study on capecitabine, oxaliplatin and bevacizumab with or without cetuximab in first‐line advanced colorectal cancer, the CAIRO2 study of the Dutch Colorectal Cancer Group (DCCG). An interim analysis of toxicity. Ann Oncol. 2008;19:734–8. 18272912 10.1093/annonc/mdm607

[44] Bajwa A , Blunt N , Vyas S , et al. Primary tumour resection and survival in the palliative management of metastatic colorectal cancer. Eur J Surg Oncol. 2009;35:164–7. 18644695 10.1016/j.ejso.2008.06.005

[45] Evans MD , Escofet X , Karandikar SS , et al. Outcomes of resection and non‐resection strategies in management of patients with advanced colorectal cancer. World J Surg Oncol. 2009;7:28. 19284542 10.1186/1477-7819-7-28 PMC2657129

[46] Ferrand F , Malka D , Bourredjem A , et al. Impact of primary tumour resection on survival of patients with colorectal cancer and synchronous metastases treated by chemotherapy: results from the multicenter, randomised trial Federation Francophone de Cancerologie Digestive 9601. Eur J Cancer. 2013;49:90–7. 22926014 10.1016/j.ejca.2012.07.006

[47] Ahmed S , Leis A , Fields A , et al. Survival impact of surgical resection of primary tumor in patients with stage IV colorectal cancer: results from a large population‐based cohort study. Cancer. 2014;120:683–91. 24222180 10.1002/cncr.28464

[48] Tsang WY , Ziogas A , Lin BS , et al. Role of primary tumor resection among chemotherapy‐treated patients with synchronous stage IV colorectal cancer: a survival analysis. J Gastrointest Surg. 2014;18:592–8. 24297651 10.1007/s11605-013-2421-0 PMC4035039

[49] Faron M , Pignon JP , Malka D , et al. Is primary tumour resection associated with survival improvement in patients with colorectal cancer and unresectable synchronous metastases? A pooled analysis of individual data from four randomised trials. Eur J Cancer. 2015;51:166–76. 25465185 10.1016/j.ejca.2014.10.023

[50] Xu H , Xia Z , Jia X , et al. Primary tumor resection is associated with improved survival in stage IV colorectal cancer: an instrumental variable analysis. Sci Rep. 2015;5:16516. 26563729 10.1038/srep16516 PMC4643284

[51] Cirocchi R , Trastulli S , Abraha I , et al. Non‐resection versus resection for an asymptomatic primary tumour in patients with unresectable stage IV colorectal cancer. Cochrane Database Syst Rev. 2012;8:Cd008997. 10.1002/14651858.CD008997.pub2 PMC11810131 22895981

